# From topography to hydrology—The modifiable area unit problem impacts freshwater species distribution models

**DOI:** 10.1002/ece3.6110

**Published:** 2020-02-21

**Authors:** Martin Friedrichs‐Manthey, Simone D. Langhans, Thomas Hein, Florian Borgwardt, Harald Kling, Sonja C. Jähnig, Sami Domisch

**Affiliations:** ^1^ Leibniz‐Institute of Freshwater Ecology and Inland Fisheries Berlin Germany; ^2^ Department of Biology Freie Universität Berlin Berlin Germany; ^3^ Department of Zoology University of Otago Dunedin New Zealand; ^4^ BC3—Basque Centre for Climate Change Leioa Spain; ^5^ Institute of Hydrobiology and Aquatic Ecosystem Management University of Natural Resources and Life Sciences Vienna Austria; ^6^ WasserCluster Lunz Lunz Austria; ^7^ Pöyry Austria GmbH Vienna Austria

**Keywords:** biomod2, catchment, Danube, fish, modifiable area unit problem, river, species distribution model

## Abstract

Species distribution models (SDMs) are statistical tools to identify potentially suitable habitats for species. For SDMs in river ecosystems, species occurrences and predictor data are often aggregated across subcatchments that serve as modeling units. The level of aggregation (i.e., model resolution) influences the statistical relationships between species occurrences and environmental predictors—a phenomenon known as the modifiable area unit problem (MAUP), making model outputs directly contingent on the model resolution. Here, we test how model performance, predictor importance, and the spatial congruence of species predictions depend on the model resolution (i.e., average subcatchment size) of SDMs. We modeled the potential habitat suitability of 50 native fish species in the upper Danube catchment at 10 different model resolutions. Model resolutions were derived using a 90‐m digital‐elevation model by using the GRASS‐GIS module *r.watershed*. Here, we decreased the average subcatchment size gradually from 632 to 2 km^2^. We then ran ensemble SDMs based on five algorithms using topographical, climatic, hydrological, and land‐use predictors for each species and resolution. Model evaluation scores were consistently high, as sensitivity and True Skill Statistic values ranged from 86.1–93.2 and 0.61–0.73, respectively. The most contributing predictor changed from topography at coarse, to hydrology at fine resolutions. Climate predictors played an intermediate role for all resolutions, while land use was of little importance. Regarding the predicted habitat suitability, we identified a spatial filtering from coarse to intermediate resolutions. The predicted habitat suitability within a coarse resolution was not ported to all smaller, nested subcatchments, but only to a fraction that held the suitable environmental conditions. Across finer resolutions, the mapped predictions were spatially congruent without such filter effect. We show that freshwater SDM predictions can have consistently high evaluation scores while mapped predictions differ significantly and are highly contingent on the underlying subcatchment size. We encourage building freshwater SDMs across multiple catchment sizes, to assess model variability and uncertainties in model outcomes emerging from the MAUP.

## Graphical abstract

Predictions of statistical species distribution models (SDMs) are dependent on the modeled spatial resolution. We show for freshwater SDMs that despite high evaluation scores, predictor importance and predicted habitat suitability can change significantly along a gradient of modeled spatial resolutions.

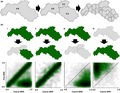

## 1 INTRODUCTION

We face a global knowledge gap regarding the actual distribution and the distribution of suitable habitats for the majority of species (Pelayo‐Villamil et al., 2015; Pimm et al., 2014), a challenge also coined as the “Wallacean shortfall” (Bini, Diniz‐Filho, Rangel, Bastos, & Pinto, 2006). Combined with the lack of resources for comprehensive species monitoring over large spatial scales (Collen, Ram, Zamin, & McRae, 2008), species distribution models (SDMs) have become an often applied tool in the field of spatial ecology and conservation biology (Carvalho, Brito, Crespo, Watts, & Possingham, 2011; Domisch et al., 2019; Franklin, 2013; Markovic et al., 2014; Ochoa‐Ochoa, Flores‐Villela, & Bezaury‐Creel, 2016; Seo, Thorne, Hannah, & Thuiller, 2009). SDMs provide range‐wide estimates of the actual and potential habitat suitability given the species data and environmental predictors at hand. SDMs can also be used in exploratory analyses by assessing environmental tolerances given the modeled predictor importance, for better understanding species abiotic requirements toward the protection and potential restoration of suitable habitats for target species (Lomolino, 2004; Porfirio et al., 2014).

In SDMs, species occurrences and predictors are statistically combined (Elith & Leathwick, 2009) to create range‐wide habitat suitability predictions. Traditionally, as SDMs have been developed in terrestrial systems (Booth, Nix, Busby, & Hutchinson, 2014), species occurrence data and environmental predictors are aggregated on gridded or hexagonal, that is, regular, modeling units (Domisch et al., 2019). The size of the modeling units (e.g., 1 km^2^) defines the model resolution and is often only limited by the availability of predictors at that given spatial resolution (Araújo, Thuiller, Williams, & Reginster, 2005; Manzoor, Griffiths, & Lukac, 2018).

**Figure 1:**
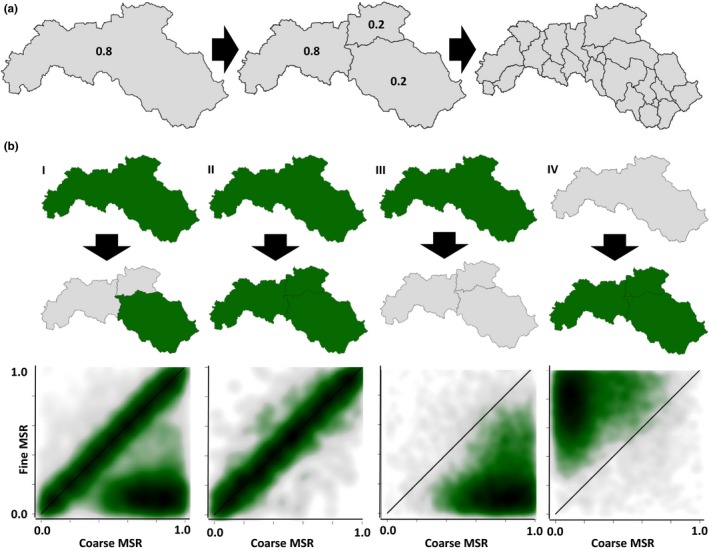
(a) Nestedness of subcatchments at different spatial resolutions. The modeled resolution increases from left to right. Additionally, the left panel and mid‐panel shows a theoretical example of predicted habitat suitability values. To analyze the spatial dependency, we correlated the theoretical value of 0.8 at the coarse resolution with all the predicted values (i.e., 0.8, 0.2, and 0.2) at the next‐finer resolution. (b) A conceptual overview of the possible outcomes of the hierarchical spatial dependency. The x‐axis represents the coarser modeled spatial resolution (MSR), while the y‐axis shows the finer resolution. Darker shading indicates a higher density of the probability values. Panel I shows a hypothetical “filter” effect. A high predicted habitat suitability at a coarse resolution results in partly high predicted suitability at a finer resolution. Panel II shows a perfect match scenario without any environmental filtering. A high suitability at a coarser resolution resulted in a high suitability in all spatially related subcatchments at a finer resolution. Panel III shows the theoretical case where a subcatchment at coarse resolution is predicted to have a high predicted habitat suitability and all the spatially related subcatchments at a finer resolution have a low predicted suitability. Panel IV shows the opposite case

In contrast to the artificial and regular modeling units used in terrestrial systems, topography‐dependent and nested modeling units are the basis for freshwater SDMs, especially for river ecosystems. To effectively include results from river SDMs into conservation management and planning, they have to be developed for catchments and subcatchments as modeling units (Domisch, Jähnig, Simaika, Kuemmerlen, & Stoll, 2015; Nel et al., 2009). Subcatchments are irregular and nonrandomly distributed units that are contingent on topography and geomorphometry (Amatulli et al., 2018). Hence, the spatial resolution of a model is the average area of subcatchments, opposed to a fixed value of for example 1 km^2^ as used for regular modeling units (see also the Pfafstetter “levels” in freshwater ecosystems; Stein, Hutchinson, & Stein, 2014). Due to the dependency of subcatchments on landscape properties, subcatchments represent a hierarchically nested system across different resolutions (Figure 1a).

The modifiable area unit problem (MAUP, Jelinski & Wu, 1996) is a common source of statistical uncertainty in spatial analyses. The MAUP occurs when statistics are performed over continuous or point data, which is aggregated on artificial spatial units. Changes in size (“scale problem”) or configuration (“zoning problem”), sensu (Jelinski & Wu, 1996) of the spatial units, are likely to change statistical relationships. Spurious or even misleading predictions can be the result. Especially in the field of conservation biology such spurious predictions can have dramatic consequences for the effectiveness of species protection and conservation measures (Moat, Bachman, Field, & Boyd, 2018).

According to the MAUP, any output of SDMs is contingent on the spatial scale and resolution, which the model is built on (Connor et al., 2018; Lobo, Jiménez‐Valverde, & Real, 2008), and estimated habitat suitability and predictor importance can vary considerably according to the spatial resolution (Connor et al., 2018; Seo et al., 2009). For instance, Seo et al. (2009) assessed range predictions for narrow, intermediate, and widely distributed plant species. They found that the predicted range sizes increased by a factor of almost three with a decrease in resolution from 1 km^2^ to 64 km^2^. Similarly, Connor et al. (2018) showed for virtual species with narrow or wide habitat niches that the range predictions for coarse resolutions (~2.5 km^2^) were up to 14 times higher compared to finer resolutions (~0.06 km^2^). Additionally, they showed that relative predictor importance for modeled, suitable habitat estimates changed from elevation to aspect (i.e., the compass direction that a slope faces) with increasing resolution. In the study from Connor et al. (2018), general model performance decreased from finer to coarser resolution. However, according to Thuiller (2003) and Swets (1988), the models from Connor et al. (2018) performed in more than 90% well, even at the coarsest resolution. In contrast, Guisan, Graham, Elith, and Huettmann (2007) found that model performance only slightly decreased when resolution decreased by a factor of 10 (100 m to 1 km, and 1 to 10 km).

Current research tries to assess the dependency of predictions on modeled spatial resolution and an assessment of uncertainty (Hui, Veldtman, & McGeoch, 2010). Hui et al. (2010) suggested to systematically change the orientation of the modeling unit or increase/decrease their size to assess how contingent identified statistical relationships are on the modeled resolution. However, despite their large importance for biodiversity conservation (McRae, Deinet, & Freeman, 2017), freshwaters and especially rivers have experienced little attention in the SDM literature (Booth et al., 2014). Despite the potentially far‐reaching implications, there are almost no studies (but see Kärcher, Frank, Walz, and Markovic (2019) for an example of resolution dependency) assessing how the MAUP is reflected in ecologically nested systems like river systems, where the modeling units are strongly dependent on topography and, consequently, their orientation and size cannot be manipulated arbitrarily.

The objectives of this study were threefold: To assess the consequences of the MAUP for SDMs on: (a) general model performance, (b) predictor importance, and on (c) predicted suitable habitats and their dependencies among resolutions (i.e., hierarchically nested subcatchments) using riverine species. We hypothesized that despite the same set of predictors and species occurrence data used: (a) general model performance will not be influenced by the model resolution (sensu Domisch, Kuemmerlen, Jähnig, & Haase, 2013), (b) predictor importance will change from a high contribution of coarse‐resolution predictors at a coarsely modeled resolution toward an increased contribution of small resolution predictors with increasing model resolution (i.e., with smaller, nested subcatchments), and that c) the predicted habitat suitability patterns will be highly dependent on the spatial resolution. In terms of hierarchically nested subcatchments, we expect that a high habitat suitability for a species is present in at least one of the next‐smaller, nested subcatchments (Figure 1bI; see Figure 1bII–IV for other theoretical possibilities).

To test our hypotheses, we modeled habitat suitability for 50 native fish species in the upper Danube catchment at 10 different spatial resolutions ranging from an average area of ~633 to ~2 km^2^, representing a wide range of resolutions that are often used for SDMs (Record et al., 2018). To assess the changes in predictor importance with changing spatial resolutions, we used a set of environmental predictors which are regularly applied in SDMs (climate, topography, land use). Additionally, we also used a hydrological time‐series as a predictor, since hydrology is known to affect the distribution of fish species (Xenopoulos & Lodge, 2006).

## 2 METHODS

### 2.1 Study area

**Figure 2:**
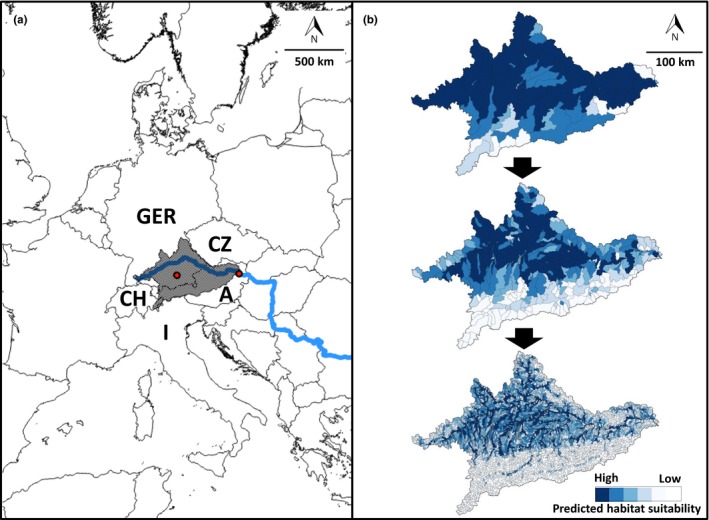
(a) Location of upper Danube catchment within Europe. Countries which have a share of the catchment are named, GER = Germany, A = Austria, I = Italy, CH = Switzerland, CZ = Czech Republic. Blue line indicates the Danube River. Red dots in Germany and Austria represent Munich and Vienna, respectively. (b) Example of bream (*Abramis brama* L.) probability of habitat suitability within the study area at three different modeled spatial resolutions (average size of subcatchments: upper panel 632 km^2^, mid‐panel 89 km^2^, lower panel 15 km^2^). The darker the blue, the more suitable is the habitat predicted

Our study was conducted in the upper Danube catchment from the source in the very Southwest of Germany up to the gauging station close to Vienna, Austria (Figure 2a). The upper Danube catchment mainly covers parts of Germany and Austria (more than 90%), as well as small parts in Switzerland, Italy, and the Czech Republic. We used the Shuttle Radar Topography Mission (SRTM) 90 m Digital Elevation Database v4.1 (http://www.cgiar-csi.org/data/srtm-90m-digital-elevation-database-v4-1) as our base digital elevation model (DEM) to extract subcatchments, i.e., the modeling units. To force the hydrological routing to follow the observed streams, we carved the vectorized European river network provided by GEOFABRIK (https://www.geofabrik.de/de/index.html) into the DEM, by a depth of 20 m. This improves the spatial representation of the observed river network, especially in topographically flat areas.

### 2.2 Defining model resolutions

We defined 10 different resolutions given the flow accumulation within the study area that is the number of upstream grid cells contributing to a river reach. We first applied the GRASS‐GIS (Neteler, Bowman, Landa, & Metz, 2012) function “*r.watershed*” (Ehlschlaeger, 1989) on the DEM, which yielded flow accumulation, drainage direction, the stream network, and the catchment delineation. We then used the “threshold” flag to define the minimum number of upstream contributing grid cells to initiate a river and thus to create subcatchments based on the flow accumulation. The lower the threshold is set, the smaller the subcatchments become. We used 10 different thresholds starting from 102,400 and ranging down to 200 grid cells, where the next‐smaller threshold was half the size of the previous threshold (i.e., 102,400, 51,200, 25,600, etc.; see Figure 2b for subcatchments created with thresholds of 102,400, 12,800, and 1,600 upstream grid cells). The spatial resolution based on the smallest threshold (200) contained the highest number of subcatchments (i.e., 162,153) and, therefore, offers the finest resolution (for details regarding the number and the average area of subcatchments, see Table 1). To cover a wider environmental gradient for each species and to avoid truncated response curves in the models (Thuiller, Brotons, Araújo, & Lavorel, 2004), we performed all calculations and models on a ~4 times larger modeling domain than the final study area (Table 1). To obtain the final predictions and results, we extracted the study region from the modeling domain. Throughout this paper, the size and number of modeling units are always given for the entire modeling domain.

**Table 1:** Overview of modeled resolutions, mean ± *SD* area of subcatchments in km^2^, and number of subcatchment for each modeled scale (modeling domain and study area)

Modeled resolution	Average size modeling domain	Subcatchments modeling domain	Average size study area	Subcatchments study area	Classification
Threshold 102,400	632 ± 781	595	714 ± 917	173	Coarse
Threshold 51,200	333 ± 400	1,130	367 ± 435	278	Coarse
Threshold 25,600	183 ± 227	2,052	201 ± 244	508	Coarse
Threshold 12,800	89 ± 102	4,207	105 ± 108	977	Intermediate
Threshold 6,400	53 ± 54	7,138	57 ± 55	1,807	Intermediate
Threshold 3,200	28 ± 29	13,292	30 ± 29	3,441	Intermediate
Threshold 1,600	15 ± 15	24,806	16 ± 14	6,608	Intermediate
Threshold 800	8 ± 8	46,594	8 ± 7	12,697	Fine
Threshold 400	4 ± 5	87,935	4 ± 4	23,983	Fine
Threshold 200	2 ± 3	162,153	2 ± 2	44,860	Fine

Note

The size and amount of subcatchments within the study area are defined by the threshold value we used in *r.watershed* function to extract the subcatchment from the digital‐elevation model.

### 2.3 Fish species data

We compiled a fish occurrence database for the upper Danube catchment combining occurrence data from five different sources. For the German part of the upper Danube catchment, we obtained fish occurrence data from the Federal Ministries of Bavaria and Baden‐Württemberg. These databases contain point occurrence data from official monitoring programs, for example, for the EU Water Framework Directive. For Austria, we used occurrence data collected within the “Improvement and Spatial extension of the European Fish Index” Project (EFI+, Pont, Bady, Logez, & Veslot, 2009). We supplemented the database with occurrence data from the Global Biodiversity Information Facility (GBIF, 2018). From GBIF, we (a) used only data collected by acknowledged sources, such as universities or federal ministries, and (b) added only occurrence records for those species that were already listed in the datasets from Bavaria, Baden‐Württemberg, or EFI+ (i.e., range filling). Similarly to how we utilized GBIF information, we used fish occurrence data from the “Digitaler Fischartenatlas von Deutschland und Österreich” (Brunken, Brunschön, Sperling, & Winkler, 2008), that is, digitized fish occurrence data collected by universities. From all data sources, we only used records spanning from 1970 to 2016. In total, we collected data for 136 fish species and removed all duplicate records per species and subcatchment at each resolution. We used occurrence information at the species level in each subcatchment per model resolution as response variable. To use an identical set of fish species across all spatial scales and to resolve a trade‐off between number of species and data availability per species, we selected those that occur in the coarsest resolution (Threshold 102,400) with at least five unique records (van Proosdij, Sosef, Wieringa, & Raes, 2016). We followed Jungwirth, Haidvogl, Hohensinner, Waidbacher, and Zauner (2014) and Schiemer and Waidbacher (1998) to define the native fish species for the upper Danube catchment and further excluded all invasive and artificially introduced species from our analyses, resulting in a total of 50 fish species (for details see Table S1).

### 2.4 Environmental data

#### 2.4.1 Climate predictors

Climate predictors were obtained from the WorldClim Version 2—Global Climate Database (http://worldclim.org/bioclim, Fick & Hijmans, 2017). WorldClim provides free climate data for ecological modeling on a ∼1 km^2^ resolution for the time period from 1970 to 2000. We initially used nine predictors describing various temperature metrics within our study region (see Table S2 for details). We calculated the average value of each predictor across each subcatchment at each spatial resolution. We aimed to use a similar time frame for all environmental predictors, and since WorldClim was the most restricted, we used the time frame from 1970 to 2000 for all environmental predictors. Note that the longer period of fish data was chosen due to data availability (an addition of almost 60% of fish records compared to 1970–2000) as well as due to the longevity of several fish species (e.g., northern pike (*Esox lucius* L.) 30 years, bream (*Abramis brama* L.) 23 years, chub (*Squalius cephalus* L.) 22 years; maximum reported age, http://www.fishbase.de, Froese, 1990).

#### 2.4.2 Land‐use predictors

Land‐use predictors were obtained from the HIstoric Land Dynamics Assessment project (HILDA, Fuchs, Herold, Verburg, & Clevers, 2012). HILDA provides global land‐use data from 1900 to 2010 on a decadal basis in five categories (forest, grassland, settlement, water, and other land) in percentage cover per grid cell. The spatial resolution of the data is ∼1 km^2^. We only used data that covered the time frame from 1970 to 2000 and calculated the mean percent coverage of each land‐use category for each subcatchment across all spatial resolutions.

#### 2.4.3 Topographical predictors

Global topographical variables were obtained from the EarthEnv project (https://www.earthenv.org/topography, Amatulli et al., 2018). We used roughness, eastness, northness, and slope at ∼1 km^2^ resolution. These data are not expected to change over the time period from 1970 to 2000. We calculated the average and range of each variable resulting in eight topographical variables (e.g., average roughness and the range of roughness across a subcatchment, Table S2).

#### 2.4.4 Hydrological predictors

Discharge data for the study region were obtained for 16 gauging stations from the German Federal Institute of Hydrology (BFG, Kling, Fuchs, & Paulin, 2012; Kling, Lagler, Stanzel, & Fuchs, 2012). The BFG provided a time‐series of monthly observed discharge data across 1970 to 2000. Discharge data from each gauging station were extrapolated to each subcatchment at each resolution using a linear relationship between observed discharge and flow accumulation. The average *R* ^2^ values for these relationships were .96 with a range from .85 to .99. Based on this extrapolation, we calculated the average annual discharge and the coefficient of variance of monthly discharge. Those two were included as predictors for the modeling procedure (Table S2).

### 2.5 Predictor preprocessing

To reduce collinearity and a potential model over‐fit (Dormann et al., 2013), we assessed the pairwise collinearity among all predictors. From an original set of 25 variables, we excluded those of the pairwise correlated variables from the analyses that had a correlation coefficient higher than |0.7|. From the remaining 14 variables, we selected two for each category (climate, land use, topography, and discharge), totaling in eight predictors for the SDMs (Table S2). All predictors were centered (so all predictors have a mean of 0) and scaled by dividing by their standard deviations. All geographical data processing was performed using the GRASS‐GIS 7.4 software (Neteler et al., 2012). Unless specified otherwise, we used the “*r.univar*” function to calculate average and range values, and “*r.mapcalc*” to perform calculations on the gridded maps.

### 2.6 Habitat suitability modeling

We built all models using the biomod2 package in R (Thuiller et al., 2016; Thuiller, Lafourcade, Engler, & Araújo, 2009) that offers the possibility to run up to 10 state‐of‐the‐art algorithms, whose predictions can be combined into a weighted ensemble model (Marmion, Parviainen, Luoto, Heikkinen, & Thuiller, 2009). The weights allow to focus on the best algorithms in the ensemble prediction, while not completely discarding results from other algorithms (Araújo & New, 2007). Furthermore, biomod2 allows setting a variety of model parameters and extracting model evaluation scores and predictor importance for every single model and the ensemble. We ran biomod2 with five algorithms, which are widely applied in the SDM literature (Merow et al., 2014): two machine‐learning algorithms (artificial neural networks, ANN; maximum entropy, MaxEnt) and three regression techniques (generalized linear model, GLM; generalized additive model, GAM; multivariate adaptive regression splines, MARS). As our fish occurrence data were presence‐only data, we had to create pseudo‐absences (Elith & Leathwick, 2009). To enable a cross‐scale comparison, we kept the ratio of pseudo‐absences relative to the number of subcatchments fixed at one third (e.g., in case of 3,000 subcatchments, we used a maximum of 1,000 pseudo‐absences). This amount was chosen to balance the need for a high amount of pseudo‐absences required for linear models, while a lower amount is required by machine‐learning techniques (Barbet‐Massin, Jiguet, Albert, & Thuiller, 2012). For each species, we selected pseudo‐absences once, randomly.

Models were evaluated by 10 separate model runs, where 70% of the data were used to calibrate the model, and 30% were used for model validation, resulting in 5,000 models (50 species × 10 repetitions × 10 spatial resolutions). For each species, model runs were combined to a weighted ensemble model, specific to each spatial resolution. Proportional weights were assigned to all single models according to the True Skill Statistic (TSS) of a given model prior to create the ensemble. The TSS values range from −1 to 1, where values close to 1 indicate a good to very good fit and values of 0 and lower indicate model performance not better than random. The assignment of weights to the models allows to automatically select the models with the best data fit for the ensemble, without completely discarding results from all other algorithms (Norberg et al., 2019). The ensemble model was then used to predict the final probabilistic habitat suitability for each species across all subcatchments at a given spatial resolution.

### 2.7 Model evaluation

We used the model sensitivity and TSS to evaluate the performance of the ensemble models. As we were using presence‐only data for the modeling approach, we chose model sensitivity as an evaluation metric, because sensitivity is the proportion of observed positives that were predicted to be positive. Sensitivity is therefore not influenced by the amount of pseudo‐absences. As a second evaluation metric, we used TSS, because it is a widely applied measure of model performance in SDM studies. However, we are aware that TSS is affected by the amount of pseudo‐absences (Allouche, Tsoar, & Kadmon, 2006).

### 2.8 Predictor importance

To analyze, which environmental predictors were mainly driving the predictions based on the 10 different spatial resolutions, we used the by default calculated predictor importance values for the ensemble model within the biomod2 package for each species at each spatial resolution. Those values range between 0 and 1 and give an estimate of how well a certain predictor correlates with the predicted habitat suitability for a species. We calculated the median value for each predictor across all 50 species at each spatial resolution.

### 2.9 Spatial congruence of predictions

We analyzed the spatial congruence between predictions at different spatial resolutions using the species‐specific suitability maps at each resolution. This was achieved by relating the predicted habitat suitability values of a species within a subcatchment at a coarser resolution to all the nested probabilistic habitat suitability values of the same species at the next‐finer resolution using linear models and by extracting the goodness of fit (Figure 1a,b). We did not translate continuous predicted probabilities into binaries (presence–absence response), because there is still discussion regarding an appropriate threshold selection (Guillera‐Arroita et al., 2015).

Data preparation (except for geographical processing), analyses, and the species distribution modeling were done using the open source software R version 3.5.2. (R Core Team, 2018).

## 3 RESULTS

### 3.1 General model performance

**Figure 3:**
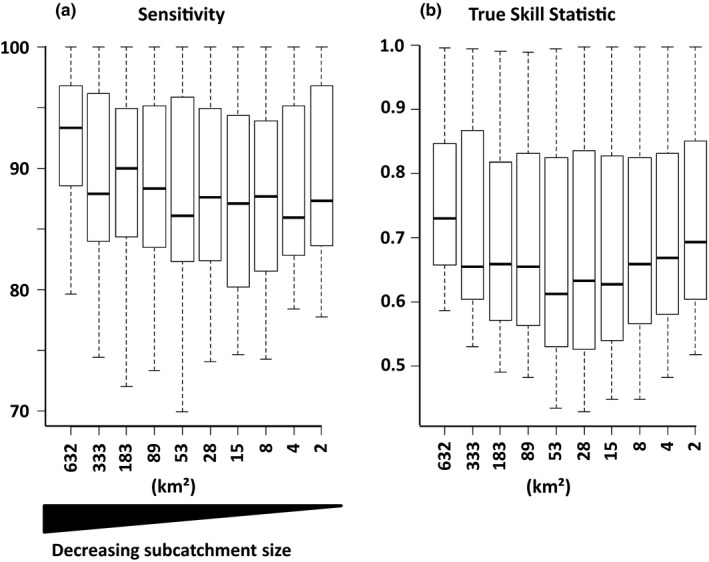
Median sensitivity (a) and median True Skill Statistic (b) values for the 50 modeled fish species in the upper Danube catchment at different modeled spatial resolutions. On the *x*‐axis, the average area of subcatchments/modeled units in square kilometers is given. Boxes indicate 1st and 3rd quantile, and whiskers indicate minimum and maximum values

The median sensitivity for all ensemble models ranged between 93.2 for the coarsest resolution (Threshold 102,400) and 86.1 for intermediate resolutions (Threshold 6,400), and sensitivity values for the models built at other spatial resolutions fell in between (Figure 3a). The median TSS values ranged from 0.73 for both the coarsest and finest resolutions to 0.61 for a intermediate resolution (Threshold 6,400). All other models ranged between those values (Figure 3b).

### 3.2 Predictor importance

**Figure 4:**
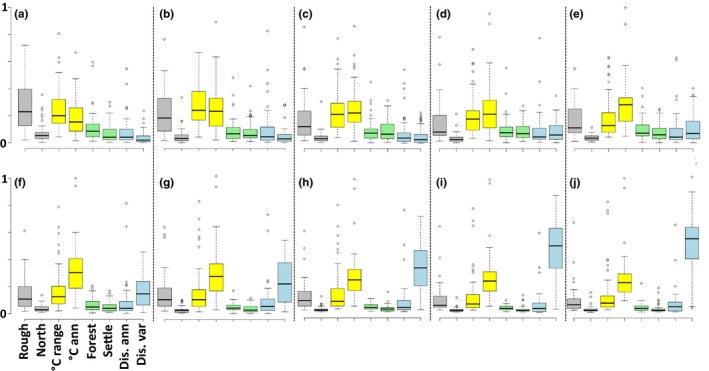
Variable importance for ensemble model predictions for 50 species in the upper Danube catchment at 10 different, modeled spatial resolutions (panels a–j are ordered from coarse spatial resolution [a = average size of the modeled subcatchment 633 km^2^] to fine spatial resolution [j = average size of the modeled subcatchment 2 km^2^]. For details, see Table 1). Colors indicate the group of predictors; from left to right: gray = topography (roughness range and average northness), yellow = climate (temperature range and average annual temperature), green = land use (average area covered by forests and mean area covered by settlements), blue = hydrology (average annual discharge and coefficient of variance of annual discharge)

We found that the median predictor importance was contingent on the spatial resolution (Figure 4). For the predictions at the coarsest resolution, topography and climate contributed most to species habitat suitability predictions (gray and yellow box‐plots, Figure 4a). With a decreasing average area of subcatchments between 333 and 15 km^2^, the relative importance of climate‐related predictors further increased (yellow box‐plots, Figure 4b–g). At fine spatial resolutions, the relative importance of hydrological predictors (blue box‐plots, Figure 4g–j) especially the coefficient of variance of monthly discharge contributed most to the predictions. Land‐use predictors (green box‐plots) were not of high importance in any of the final ensemble models.

### 3.3 Spatial dependency across spatial resolutions

**Figure 5:**
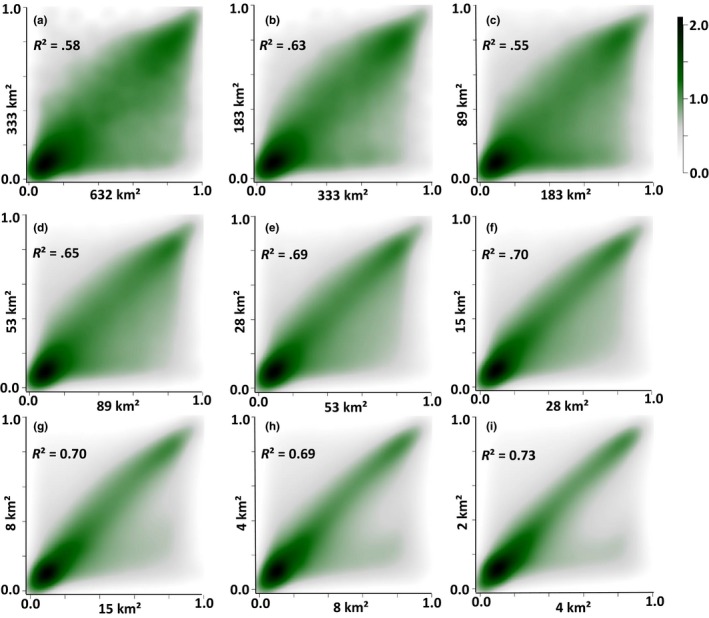
Relationship between predicted summed probabilities for each subcatchment at a lower modeled spatial resolution (*x*‐axis) and the highest predicted probability for the spatially related subcatchment at the next‐finer modeled spatial resolution (*y*‐axis). Axis labels indicate the average size of modeled subcatchments. Panels are ordered from coarse to fine resolutions

For all combinations of a coarse versus the next‐finer spatial resolution (Figure 5), linear models revealed a significant positive relationship between the predicted habitat suitability of a coarser resolution and that of the next‐finer resolution. Although all the identified relationships were positive, we found an increase in *R* ^2^ values the higher the modeled resolution gets (Figure 5). Especially for coarse resolutions (Figure 5a–c), we found lower *R* ^2^ values when comparing predicted habitat suitability between related modeled resolutions. Almost all nonmatching probabilities (i.e., pronounced difference between predicted probability at coarser resolution with predicted probability at next‐finer resolution) were below the regression line indicating a pronounced “filter effect”, meaning that only a fraction of coarse‐resolution subcatchments were suitable in the nested (i.e., next‐finer resolution) subcatchments (Figure 5a–f, dark green area in the lower right corner). For fine resolutions, the *R* ^2^ values increased, indicating that this “filtering effect” was less pronounced, for example habitat suitability predictions did not differ between a subcatchment at a coarser resolution and those that are nested within the next‐finer resolution (Figure 5g–I).

## 4 DISCUSSION

Using a weighted ensemble SDM approach, we analyzed the effects of 10 different spatial resolutions on the general model performance, predictor importance, and the contingency on modeled resolution of predicted habitat suitability for 50 native fish species in the upper Danube catchment. The general model performance remained high across all spatial resolutions, whereas predictor importance was contingent on the spatial resolution (i.e., average subcatchment size). Additionally, we identified strong spatial dependencies in the mapped habitat suitability patterns across spatial resolutions.

### 4.1 General model performance

For all spatial resolutions, model performance measures—sensitivity as well as TSS—were high to very high (Figure 3), indicating the validity and applicability of the respective model (Swets, 1988; Thuiller, 2003). This finding confirms our first hypothesis and is of special interest when considering that models at coarse, intermediate, and fine resolutions were mainly driven by topography, climate, or hydrology, respectively (Figure 4). The constantly high model performance over all modeled resolutions with changing predictor importance suggests that, at a given spatial resolution, the model uses the most informative environmental predictors that increase the proportion of explained variance regarding the distribution of suitable habitats. As we let the model “decide” which predictors are most useful (opposed to a preselection of predictors), it remains to be assessed how a strictly defined set of predictors at each spatial resolution would impact model outcomes (Synes & Osborne, 2011). We hypothesize that a further increase in model resolution would call for a new set of for example hydraulic predictors at even finer spatial resolutions, since our hydrological predictors would not be able to reflect such patterns.

### 4.2 Predictor importance

The importance of topographical, climatic, and hydrological predictors on model outputs was dependent on the given spatial resolution. Models at coarse resolutions were mainly driven by topography, climate was dominating the intermediate resolutions, and hydrology was found to mainly affect predictions at fine spatial resolutions. This finding confirms our second hypothesis. However, we argue that this finding is directly linked to the MAUP and is not a species or organism specific result. Most likely the aggregation of predictor values (1 km^2^ grid cell values) across modeled subcatchments at each spatial resolution is causing the observed change in predictor importance. For 30 of the 50 studied fish species, most suitable habitats were located in lowland regions. In lowland regions, we find only small gradients of topography and climate across large areas. Consequently, the spatial aggregation of topography and climate predictors within each subcatchment yield only minor changes compared to the original values. Hydrology (i.e., the coefficient of variation of annual discharge), however, can be highly variable across “flat” regions if measured across multiple stream orders. During the spatial aggregation process, this variability in hydrology flattened and resulted in an overall weak contribution in model predictions.

Land use‐related predictors, which are often identified as one of the main factors driving habitat suitability predictions of fish, besides climate (Radinger et al., 2016), did not play an important role in any of the modeled resolutions. We argue that the strong gradients within the other predictors (e.g., topography and climate from alpine to lowland, and hydrology from very small streams to the main channel) may mask the effects of the rather broad land‐use categories used in our analysis (Feld et al., 2016; Manzoor et al., 2018).

By using a variety of model resolutions, we were able to show a very general problem for freshwater SDM studies with far‐reaching implications: The modeled spatial resolution affects predictor importance. In most SDMs, the choice of predictors is, besides being ecologically meaningful, driven by their availability (Booth et al., 2014), while the model resolution is often chosen according to the resolution of the predictor and species occurrence data (Manzoor et al., 2018). Our results highlight that conclusions drawn from SDM outputs built at a specific spatial resolution may be seen as a “spatial snap shot” and are only valid for the specific resolution used. Hence, such results need to be interpreted carefully, even when the SDM performs well in terms of evaluation scores.

### 4.3 Predicted habitat suitability correlated between resolutions

Spatially related subcatchments and their predicted species‐specific suitability values across different resolutions were strongly correlated. Interestingly, this pure statistical finding reveals strong parallels to the landscape filter hypothesis (Poff, 1997). According to the landscape filter hypothesis, an organism has to pass several environmental filters to thrive at the location where it is observed. In line with the landscape filter hypothesis, we found that a subcatchment with a high predicted habitat suitability at a coarse resolution was at least partly suitable at the next‐finer resolution (i.e., only when a species is able to pass a filter at a coarse resolution, it can occur in parts of this area at finer resolutions). The statistical possible, but ecological impossible opposite case was only rarely observed. This “filter effect” (Figure 5, green area in the lower right corner of each panel) was less pronounced at finer spatial resolutions, indicating a tipping point of the spatial resolution beyond which the filter effect did not apply anymore given the chosen predictors. It remains to be tested whether using even smaller subcatchments (and, e.g., hydrological variables over shorter time periods) would lead to a continuation of this pattern. Given the predictors set, we could not identify the opposite case of such a filter effect, that is where a low habitat suitability at a coarse resolution would turn into a high suitability in the nested subcatchments.

### 4.4 Implications for spatial conservation planning

Despite that statistical SDMs and the evaluation scores only assess the mathematical correctness of the model and do not provide any information on the ecological meaningfulness (Domisch et al., 2013; Mouton, De Baets, & Goethals, 2010), SDMs are regularly used for conservation purposes (Record et al., 2018). Our finding that predictor importance—a measure often used to inform conservation management (Lomolino, 2004; Porfirio et al., 2014)—and predictions itself are contingent on the modeled spatial resolution is important, for example, when SDM outputs inform decisions regarding the delineation of protected areas (Loiselle et al., 2003) or the assessment of species' environmental habitat preferences (e.g., Mantyka‐Pringle, Martin, Moffatt, Linke, & Rhodes, 2014; Markovic et al., 2014; Radinger et al., 2016). Any output derived from an SDM should be evaluated in context of the chosen predictors and the spatial resolution at which the model is built. According to the MAUP, and in the absence of the possibility to change orientation and/or configuration of modeling units (Hui et al., 2010), our study highlights the necessity of performing catchment‐based river SDMs at multiple spatial resolutions to assess uncertainties and/or range in predictor importance and predictions made. This is of special interest considering that model performance measures are not affected by the MAUP and, consequently, do not reflect those uncertainties. Without considering the MAUP, any generalization about habitat suitability and predictor importance beyond specific spatial resolutions, at which the model has been built, have to be formulated and/or used carefully.

### 4.5 Our results in a practical context

Hydrology was the main driver of habitat suitability predictions for fish species at fine resolutions in our study. Hence, fine‐scale predictions are required when SDM outputs should support conservation management in freshwater ecosystems (Groves et al., 2002). The increasing availability of high‐resolution predictors at global scale (e.g., Domisch, Amatulli, & Jetz, 2015, and especially hydrology; Barbarossa et al., 2018) is promising to improve the accuracy needed to use SDMs for the on‐ground implementation of conservation measures. By only using the results from our fine resolution model, protecting morphological intact stream stretches and restoring a natural flow regime may seem to be appropriate measures to create suitable habitats for the majority of fish species in this area. However, as expected by the MAUP, this strong statistical relationship is contingent on the modeled spatial resolution. As a consequence, only applying the fine resolution model, climate would not be important for habitat suitability estimates in our study region. In contrast, with similar performance, our models on intermediate spatial resolutions identified climate as the main driver for habitat suitability predictions. Hence, the spatial allocation of conservation measures and protected areas should take the advantage of this “filter effect,” focusing on climatically suitable areas, although climate has not been identified as important for the fine resolution models. If the potentially protected or restored sites would be located in climatically unsuitable areas, either today or in the near future, it is likely that they would not be successful in supporting a healthy fish fauna.

## AUTHOR CONTRIBUTIONS

SD, SCJ, SDL and MF‐M conceived the research; MF‐M and SD did the modeling; all authors drafted the manuscript and all authors gave final approval for publication.

## Data Availability

All results supporting the study will be available at PANGAEA (https://www.pangaea.de/; https://doi.org/10.1594/PANGAEA.911422).
