# Supplementary material for: From topography to hydrology—The modifiable area unit problem impacts freshwater species distribution models
Source: Ecol Evol. 2020 Feb 21;10(6):2956–68. doi: 10.1002/ece3.6110 (PMC7083667; doi:10.1002/ece3.6110)
Supplement: Supplementary file 1 [file ECE3-10-2956-s001.pdf]

1 Supplementary Material

Table S1: All 50 native species modeled and the number of occurrences and proportion of sub-catchments with known occurrences (in brackets) for each spatial resolution. T. = Threshold

Species	T. 102400	T. 51200	T. 25600	T. 12800	T. 6400	T. 3200	T. 1600	T. 800	T. 400	T. 200
Abramis brama	135 (0.23)	215 (0.19)	283 (0.14)	385 (0.09)	477 (0.07)	566 (0.04)	648 (0.03)	704 (0.02)	758 (0.01)	800 (<0.01)
Alburnoides bipunctatus	135 (0.23)	220 (0.19)	296 (0.14)	373 (0.09)	460 (0.06)	532 (0.04)	618 (0.02)	698 (0.01)	756 (0.01)	814 (0.01)
Alburnus alburnus	147 (0.24)	245 (0.22)	327 (0.16)	428 (0.10)	536 (0.08)	641 (0.05)	755 (0.03)	839 (0.02)	915 (0.01)	965 (0.01)
Aspius aspius	85 (0.14)	118 (0.10)	139 (0.07)	171 (0.04)	192 (0.03)	207 (0.02)	218 (0.01)	225 (<0.01)	235 (<0.01)	240 (<0.01)
Ballerus sapa	31 (0.05)	36 (0.03)	39 (0.02)	44 (0.01)	48 (0.01)	50 (<0.01)	51 (<0.01)	52 (<0.01)	55 (<0.01)	56 (<0.01)
Barbatula barbatula	165 (0.27)	308 (0.27)	471 (0.23)	691 (0.16)	951 (0.13)	1239 (0.09)	1477 (0.06)	1693 (0.04)	1846 (0.02)	1988 (0.02)
Barbus balcanicus	20 (0.03)	31 (0.03)	37 (0.02)	42 (0.01)	51 (0.01)	59 (<0.01)	74 (<0.01)	79 (<0.01)	80 (<0.01)	85 (<0.01)
Barbus barbus	161 (0.27)	277 (0.25)	377 (0.18)	509 (0.12)	619 (0.09)	721 (0.05)	836 (0.03)	939 (0.02)	1029 (0.01)	1110 (0.01)
Blicca bjoerkna	85 (0.14)	119 (0.11)	146 (0.07)	191 (0.05)	222 (0.03)	254 (0.02)	289 (0.01)	303 (0.01)	319 (<0.01)	328 (<0.01)
Carassius auratus	62 (0.10)	75 (0.07)	92 (0.04)	119 (0.03)	129 (0.02)	142 (0.01)	156 (0.01)	164 (<0.01)	176 (<0.01)	180 (<0.01)
Carassius carassius	114 (0.19)	163 (0.14)	197 (0.10)	247 (0.06)	287 (0.04)	328 (0.02)	363 (0.01)	386 (0.01)	405 (<0.01)	418 (<0.01)
Carassius gibelio	124 (0.21)	203 (0.18)	251 (0.12)	320 (0.08)	372 (0.05)	404 (0.03)	434 (0.02)	447 (0.01)	461 (0.01)	474 (0.01)
Chondrostoma nasus	153 (0.26)	243 (0.22)	327 (0.16)	429 (0.10)	509 (0.07)	577 (0.04)	647 (0.03)	706 (0.02)	760 (0.01)	798 (0.01)
Cobitis elongatoides	45 (0.08)	70 (0.06)	82 (0.04)	93 (0.02)	107 (0.01)	121 (0.01)	129 (0.01)	137 (<0.01)	144 (<0.01)	148 (<0.01)
Cobitis taenia	62 (0.10)	82 (0.07)	112 (0.05)	173 (0.04)	225 (0.03)	279 (0.02)	325 (0.01)	376 (0.01)	414 (<0.01)	447 (<0.01)
Cottus gobio	197 (0.33)	339 (0.30)	534 (0.26)	853 (0.20)	1201 (0.17)	1577 (0.12)	1958 (0.08)	2267 (0.05)	2558 (0.03)	2774 (0.03)
Ctenopharyngodon idella	30 (0.05)	33 (0.03)	33 (0.02)	33 (0.01)	33 (<0.01)	34 (<0.01)	34 (<0.01)	34 (<0.01)	35 (<0.01)	36 (<0.01)
Cyprinus carpio	184 (0.31)	282 (0.25)	379 (0.18)	538 (0.13)	632 (0.09)	734 (0.06)	837 (0.03)	901 (0.02)	965 (0.01)	1008 (0.01)
Esox lucius	202 (0.34)	327 (0.29)	470 (0.23)	667 (0.16)	880 (0.12)	1105 (0.08)	1305 (0.05)	1479 (0.03)	1624 (0.02)	1732 (0.02)
Eudontomyzon mariae	32 (0.05)	42 (0.04)	59 (0.03)	73 (0.02)	80 (0.01)	98 (0.01)	111 (<0.01)	117 (<0.01)	122 (<0.01)	124 (<0.01)
Gobio obtusirostris	201 (0.34)	350 (0.31)	530 (0.26)	775 (0.18)	1044 (0.15)	1339 (0.10)	1598 (0.06)	1812 (0.04)	1968 (0.02)	2126 (0.02)
Gymnocephalus baloni	21 (0.04)	23 (0.02)	27 (0.01)	36 (0.01)	37 (0.01)	40 (<0.01)	40 (<0.01)	41 (<0.01)	43 (<0.01)	43 (<0.01)
Gymnocephalus cernua	77 (0.13)	97 (0.09)	111 (0.05)	143 (0.03)	160 (0.02)	170 (0.01)	178 (0.01)	185 (<0.01)	190 (<0.01)	191 (<0.01)
Gymnocephalus schraetser	27 (0.05)	31 (0.03)	33 (0.02)	37 (0.01)	39 (0.01)	41 (<0.01)	42 (<0.01)	43 (<0.01)	45 (<0.01)	45 (<0.01)
Hucho hucho	64 (0.11)	79 (0.07)	105 (0.05)	121 (0.03)	149 (0.02)	163 (0.01)	183 (0.01)	197 (<0.01)	209 (<0.01)	219 (<0.01)
Lampetra planeri	53 (0.09)	80 (0.07)	110 (0.05)	133 (0.03)	160 (0.02)	171 (0.01)	182 (0.01)	193 (<0.01)	198 (<0.01)	203 (<0.01)

Table S1: All 50 native species modeled and the number of occurrences and proportion of sub-catchments with known occurrences (in brackets) for each spatial resolution. T. = Threshold *(continuation)*

**Species**	**T. 102400**	**T. 51200**	**T. 25600**	**T. 12800**	**T. 6400**	**T. 3200**	**T. 1600**	**T. 800**	**T. 400**	**T. 200**
Leucaspius delineatus	42 (0.07)	61 (0.05)	70 (0.03)	91 (0.02)	106 (0.01)	118 (0.01)	122 (<0.01)	129 (<0.01)	131 (<0.01)	135 (<0.01)
Leuciscus idus	90 (0.15)	135 (0.12)	171 (0.08)	220 (0.05)	254 (0.04)	279 (0.02)	302 (0.01)	318 (0.01)	335 (<0.01)	345 (<0.01)
Leuciscus leuciscus	150 (0.25)	268 (0.24)	389 (0.19)	551 (0.13)	729 (0.10)	908 (0.07)	1054 (0.04)	1186 (0.03)	1286 (0.01)	1366 (0.01)
Lota lota	129 (0.22)	189 (0.17)	249 (0.12)	333 (0.08)	409 (0.06)	469 (0.04)	506 (0.02)	539 (0.01)	568 (0.01)	591 (0.01)
Misgurnus fossilis	33 (0.06)	41 (0.04)	47 (0.02)	52 (0.01)	55 (0.01)	63 (<0.01)	71 (<0.01)	76 (<0.01)	82 (<0.01)	89 (<0.01)
Perca fluviatilis	204 (0.34)	349 (0.31)	502 (0.24)	716 (0.17)	927 (0.13)	1152 (0.09)	1345 (0.05)	1489 (0.03)	1603 (0.02)	1692 (0.02)
Phoxinus phoxinus	156 (0.26)	265 (0.23)	374 (0.18)	539 (0.13)	670 (0.09)	810 (0.06)	946 (0.04)	1078 (0.02)	1194 (0.01)	1293 (0.01)
Rhodeus amarus	112 (0.19)	182 (0.16)	237 (0.12)	308 (0.07)	365 (0.05)	418 (0.03)	464 (0.02)	501 (0.01)	528 (0.01)	555 (0.01)
Romanogobio kesslerii	16 (0.03)	22 (0.02)	24 (0.01)	24 (0.01)	27 (<0.01)	29 (<0.01)	32 (<0.01)	33 (<0.01)	36 (<0.01)	38 (<0.01)
Romanogobio vladykovi	63 (0.11)	82 (0.07)	95 (0.05)	110 (0.03)	123 (0.02)	133 (0.01)	140 (0.01)	147 (<0.01)	155 (<0.01)	156 (<0.01)
Rutilus meidingeri	18 (0.03)	27 (0.02)	29 (0.01)	31 (0.01)	38 (0.01)	42 (<0.01)	43 (<0.01)	45 (<0.01)	49 (<0.01)	51 (<0.01)
Rutilus rutilus	180 (0.30)	318 (0.28)	481 (0.23)	726 (0.17)	955 (0.13)	1213 (0.09)	1438 (0.06)	1624 (0.03)	1776 (0.02)	1901 (0.02)
Rutilus virgo	15 (0.03)	21 (0.02)	24 (0.01)	29 (0.01)	31 (<0.01)	33 (<0.01)	40 (<0.01)	40 (<0.01)	42 (<0.01)	45 (<0.01)
Salmo trutta	186 (0.31)	359 (0.32)	614 (0.30)	1041 (0.25)	1585 (0.22)	2266 (0.17)	2963 (0.12)	3479 (0.07)	3964 (0.05)	4359 (0.05)
Sander lucioperca	99 (0.17)	149 (0.13)	189 (0.09)	242 (0.06)	274 (0.04)	304 (0.02)	338 (0.01)	353 (0.01)	372 (<0.01)	386 (<0.01)
Sander volgensis	6 (0.01)	7 (0.01)	8 (<0.01)	8 (<0.01)	9 (<0.01)	9 (<0.01)	9 (<0.01)	9 (<0.01)	9 (<0.01)	9 (<0.01)
Scardinius erythrophthalmus	184 (0.31)	280 (0.25)	375 (0.18)	526 (0.13)	653 (0.09)	771 (0.06)	890 (0.04)	974 (0.02)	1048 (0.01)	1088 (0.01)
Silurus glanis	116 (0.19)	158 (0.14)	193 (0.09)	239 (0.06)	268 (0.04)	298 (0.02)	322 (0.01)	334 (0.01)	348 (<0.01)	360 (<0.01)
Squalius cephalus	67 (0.11)	104 (0.09)	159 (0.08)	242 (0.06)	332 (0.05)	431 (0.03)	522 (0.02)	610 (0.01)	667 (0.01)	719 (0.01)
Telestes souffia	31 (0.05)	40 (0.04)	52 (0.03)	65 (0.02)	76 (0.01)	85 (0.01)	95 (<0.01)	99 (<0.01)	107 (<0.01)	116 (<0.01)
Tinca tinca	183 (0.31)	285 (0.25)	393 (0.19)	571 (0.14)	723 (0.10)	887 (0.07)	1037 (0.04)	1148 (0.02)	1245 (0.01)	1319 (0.01)
Vimba vimba	62 (0.10)	80 (0.07)	99 (0.05)	109 (0.03)	126 (0.02)	135 (0.01)	145 (0.01)	151 (<0.01)	159 (<0.01)	161 (<0.01)
Zingel streber	36 (0.06)	46 (0.04)	57 (0.03)	67 (0.02)	73 (0.01)	77 (0.01)	83 (<0.01)	87 (<0.01)	92 (<0.01)	93 (<0.01)
Zingel zingel	25 (0.04)	29 (0.03)	35 (0.02)	41 (0.01)	45 (0.01)	46 (<0.01)	48 (<0.01)	50 (<0.01)	54 (<0.01)	54 (<0.01)

Table 2: Overview of variables used to model the distribution of fish species in the Upper Danube catchment. We show the full set of variables, the ones which were not correlated and the final set of variables used in the models

**Initial Variable**	**Not correlated**	**Used in the models**	**Category**	**Source**
Annual Average Temperature	+	+	climate	BIOCLIM
Average Diurnal Range			climate	BIOCLIM
Temperature Seasonality			climate	BIOCLIM
Maximum Temperature of Warmest Month			climate	BIOCLIM
Temperature Annual Range	+	+	climate	BIOCLIM
Average Temperature of Wettest Quarter	+		climate	BIOCLIM
Average Temperature of Driest Quarter	+		climate	BIOCLIM
Average Temperature of Warmest Quarter			climate	BIOCLIM
Average Temperature of Coldest Quarter			climate	BIOCLIM
Average Area covered by Forest	+	+	land use	HILDA
Average Area covered by Grassland	+		land use	HILDA
Average Area covered by Settlement	+	+	land use	HILDA
Average Area covered by Water	+		land use	HILDA
Average Area covered by other Land	+		land use	HILDA
Average roughness			topography	EarthEnv
Range roughness	+	+	topography	EarthEnv
Average Eastness	+		topography	EarthEnv
Range Eastness			topography	EarthEnv
Average Northness	+	+	topography	EarthEnv
Range Northness			topography	EarthEnv
Average slope			topography	EarthEnv
Range slope			topography	EarthEnv
Average annual discharge	+	+	hydrology	BfG
Coefficient of variance of monthly discharge	+	+	hydrology	BfG
